# Encapsulation of *Satureja khuzistanica* extract in alginate hydrogel accelerate wound healing in adult male rats

**DOI:** 10.1186/s41232-019-0090-4

**Published:** 2019-01-30

**Authors:** Fatemeh Beyranvand, Ahmad Gharzi, Abolfazl Abbaszadeh, Reza Mohammadrezaei Khorramabadi, Mohammadreza Gholami, Anneh Mohammad Gharravi

**Affiliations:** 1 Department of Biology, School of Basic Sciences, Lorestan University, Khorramabad, Iran; 2 Department of Biology, School of Basic Sciences, Razi University, Kermanshah, Iran; 3 Razi Herbal Medicines Research Center and Department of Surgery, Lorestan University of Medical Sciences, Khorramabad, Iran; 4 Student Research Committee, Lorestan University of Medical Sciences, Khorramabad, Iran; 5 Department of Anatomical Sciences, Faculty of Medicine, Kermanshah University of Medical Sciences, Kermanshah, 67148-69914 Iran; 6 Tissue Engineering and Stem Cells Research Center, Shahroud University of Medical Sciences, Shahroud, Iran

**Keywords:** Hydrogel alginate, *Satureja khuzistanica*, Wound healing, Skin wound

## Abstract

**Background:**

Finding the best dressing for a specific *wound* had continued from the past to present. The aim of this study was to evaluate the *effect* of encapsulated extract of *Satureja khuzistanica* in hydrogel alginate at wound healing.

**Methods:**

Thirty-two male Wistar rats with a puncture wound in the back of the neck skin were divided randomly into four groups including a control group, *Satureja khuzistanica*-treated group, hydrogel alginate-treated group, and *Satureja khuzistanica* encapsulated in hydrogel alginate-treated group. Rats were treated for 22 days. The skin samples were taken on 3rd, 7th, 14th, and 22nd days after treatment for light microscopy. Results were analyzed in accordance with Kruskal-Wallis and Friedman test (for histopathology analysis) by using SPSS v.22 software.

**Results:**

Macroscopically evaluations and measurement of wound size showed increased wound healing process in the treated groups. The complete improvement was created on the 14th day. The wound site was not observed on the 22nd day. But the wound site was observed on the 22nd day in the control group. Also, comparison of the percentage of wound healing between the treated and control groups on 3rd, 7th, 14th, and 22nd days showed a significant difference (*p* < 0.05). Comparison of the H&E stained sections in the studied groups showed that treated groups were effective on wound healing in comparison with the control group.

**Conclusions:**

Encapsulated extract of *Satureja khuzistanica* in hydrogel alginate may accelerate wound improvement and increase the rate of wound healing without scar formation.

## Background

One of the widest and heaviest organs of the body is the skin; it includes about 15% of the body weight. Many biological functions are associated with skin, such as the creation of a protective barrier against environmental invaders (physical, chemical, and biological factors), regulation of humidity of the body by preventing water loss, and thermoregulation of the body. In addition, skin is a vital member of the integumentary system. Three layers form the skin structure including the epidermis (the outer layer), the dermis, and the subcutaneous tissue (the inner layer) [1, 2]. Therefore, impairment in the continuity of the skin because of injury or illness can cause irreparable damages such as disability and death. [3]. Wound is a kind of damage to the integrity of skin that is occurred subsequent a trauma or diseases [4]. The wounds are classified conventionally in accordance with their depth (the superficial and deep) and the length of wound healing process (acute and chronic) [5]. Therefore, the healing of the wound as the main process is involved in the regeneration of tissue. The repair of wound is an interactive and continuous mechanism that includes three overlapping events such as inflammatory, proliferative, and tissue remodeling. Keratinocytes, inflammatory cells, extracellular matrix (ECM), cytokines, growth factors, and other mediators are the cellular and biochemical components that participate in the wound healing process [6, 7]. The first phase of repair is initiated by inflammation. In the inflammatory phase, leukocytes infiltrate to the wound site and secrete different mediators and eliminate the dead cells, subsequently. Stimulating factors produced by the inflammatory cells create the proliferative phase. Tissue regeneration is the most important event that occurred during this stage. Regeneration is developed in response to the previous phase. Eventually, tissue remodeling as the latest phase of wound healing is developed. In this phase, vessels repair and the extracellular matrix reorganize [8–10]. So, defects in the wound healing process can lead to morbidity and mortality [11]. In fact, wound healing is a process that depends on wound dressings as an important part of wound management. Before, wound dressing was done by using natural products such as different kinds of gauzes and plant preparation including grape seed, lemon, rosemary, and jojoba. [6, 12]. But today, biomaterials are recognized as new candidates for wound dressing [13].

Some of these materials which form tridimensional hydrophilic networks are hydrogels. They are able to absorb water in fluids without solvability and any changes in its structure. Natural hydrogels can act as protective agents for maintaining moisture and play a role in wound healing. Combination of hydrogels with natural polymers can be used in drug delivery systems by improving the effects of therapeutic agents. The source of these materials is the nature, and some of them, such as alginate and collagen, are used in medicine because of their structure. Alginate is a natural-origin anionic polymer which is abundantly found in algae especially in brown seaweeds. It is widely used in medicine because of its advantages, e.g., compatibility, low price, and similar structure with the body tissues. Alginate hydrogels are a kind of hydrogels based on natural polymers that are similar to extracellular matrices (ECM) so that they can participate in the wound healing process by using drug delivery. Therefore, alginate gels can play roles in functional improvement and release of bioactive compounds of drugs or plant materials in the wound site [14–17].

*Satureja khuzistanica* is categorized as an annual plant that is a member of the family Lamiaceae (mint family). *S. khuzistanica* is a native plant of Iran that is geographically distributed in the south and west of this country. Various studies approved the biological properties of different parts of this plant including antiviral (anti-HIV-1), antibacterial, anti-inflammatory, antispasmodic, anti-nociceptive, antifungal, antioxidant, antiseptic, antidiarrheal, antiprotozoal, and vasodilator effects. [18–21].

So, according to the beneficial role of natural polymers in the treatment of disease, the aim of this study was to evaluate the *effect* of encapsulated extract of *Satureja khuzistanica* in alginate hydrogel at wound healing in adult male Wistar rats.

## Methods

### Chemicals

The following chemicals including ethanol (Merck Company, Germany), paraffin, eosin stain, hematoxylin stain, xylene (Merck Company, Germany), formalin (Merck Company, Germany), acetic acid (Merck Company, Germany), ketamine HCl, xylazine, alginate powder, NaCl (Merck Company, Germany), distilled water, and HCl (Merck Company, Germany) were purchased.

### Preparation of plant material

Aerial parts of *Satureja khuzistanica* (leaves and stem) were obtained from the Khorraman Company in Lorestan province. These parts were dried in the shadow at room temperature. Then, plant materials were powdered.

### Extract preparation

The preparation of extract was carried out based on the maceration method. According to this method, 700 g of plant powder was mixed with 1-l methanol (98%). Then, the solution was placed in the laboratory for 2 days. After that, filtration of solution and concentration of it were performed by using a filter paper and rotary evaporator, respectively [18, 22].

### Alginate solution preparation

Twenty millimolar HEPES and 150 mM NaCl were dissolved in distilled water and were heated to a temperature of 60 °C. Then, 1.25 g of alginate powder was added to the above solution and subsequently was placed on a stirrer for 1–2 h. After that, the solution was maintained at room temperature and the solution was adjusted to pH = 7.4. Eventually, by adding distilled water, it was brought to volume.

### Alginate hydrogel dressing preparation

The drops of the above solution were added slowly to a sterile container containing calcium chloride. Then, it was kept in this solution for about 10–15 min until alginate beads are completely polymerized. Finally, washing in NaCl was carried out.

### Encapsulated *Satureja khuzistanica* extract in alginate hydrogel dressing preparation

First, the *Satureja khuzistanica* ethanolic extract was diluted by ethanol (5 ml ethanol per gram of dry matter). Then, it was added to the alginate hydrogel dressing solution. The obtained solution was kept at room temperature for 1 day in order to complete the encapsulation of *Satureja khuzistanica* extract in alginate [14].

### Animals and study design

This study was performed in Razi Herbal Medicine Research Center of the Lorestan University of Medical Sciences. All of the 32 male Wistar rats (weighing 150–180 g) were obtained from the Razi Herbal Medicine Center of Lorestan University of Medical Sciences. For adaptation of rats, they were placed in the animal laboratory for 1 week at 22 °C. Suitable environmental condition including 12 h light and 12 h dark cycle and free access to standard rat food and water were prepared for the rats. The rats were separated into four groups (*n* = 8) as follows:

- Group 1: Control group (wound without treatment)
- Group 2: *Satureja khuzistanica*-treated group (wound treated with ethanolic extract of *Satureja khuzistanica* (1 g per day))
- Group 3: Alginate hydrogel dressing-treated group (surgery along with alginate hydrogel dressing treatment)
- Group 4: Alginate hydrogel/*Satureja khuzistanica* dressing-treated group (wound along with alginate hydrogel dressing and ethanolic extract of *Satureja khuzistanica* treatment)

### Surgical procedure

First, the rats were prepared for surgery with an injection of ketamine HCl (50 mg/kg) and xylazine (5 mg/kg) intraperitoneally for anesthetization. After that, the surface of the skin was shaved and disinfected. Then, the wound was cut in the form of 1 cm circular diameter by using biopsy punch in the three layers of the skin (dermis, epidermis, and hypodermis). After that, immediate treatment was started. Finally, the skin of the wound site was removed at the 3rd day, 7th day, 14th day, and 22nd day after surgery for histopathologic examinations. Then, the skins were placed in 10% formalin for fixation [23].

### Histopathologic studies

The following steps were conducted after fixation in 10% formalin: embedding in paraffin and cutting of sections with a microtome (4–6-μm-thick). Then, graded ethanol series and xylene were used for dehydration and clearing, respectively. After that, hematoxylin-eosin staining (H&E) was conducted. Section study was done according to Asadi et al., by light microscopy method. Different parameters including epithelial regeneration, granulation tissue thickness, number of fibroblasts, macrophages, and neutrophils, and angiogenesis assessment of wound site were measured in order to the rank of wound healing [6].

### Statistical analysis

Results were analyzed in accordance with the Kruskal-Wallis and Friedman tests (for histopathology analysis) by using SPSS v.22 software. Values were expressed statistically significant at *p* < 0.05.

## Results

### Light microscopic findings on the third day

Section study on the third day of treatment showed that wound healing and epidermis formation has started in the *Satureja khuzistanica*-treated wounds, alginate hydrogel dressing-treated groups, alginate hydrogel/*Satureja khuzistanica* dressing-treated wounds, and even in the control group wounds. The section study showed that the formation of blood vessels has been initiated in the *Satureja khuzistanica*-treated wounds, alginate hydrogel dressing-treated groups, alginate hydrogel/*Satureja khuzistanica* dressing-treated wounds, and control group wounds, but this process of formation of blood vessels in alginate hydrogel groups was more than that in the other groups. Also, the formation of granulation has started in the *Satureja khuzistanica*-treated wounds, alginate hydrogel dressing-treated groups, alginate hydrogel/*Satureja khuzistanica* dressing-treated wounds, and control group, but the granulation in the alginate hydrogel/*Satureja khuzistanica* dressing-treated wounds was more than that in the other groups.

### Light microscopic findings on the seventh day

Results of evaluation on the seventh day of treatment identified that the process of wound healing and epidermis formation has progressed in the *Satureja khuzistanica*-treated wounds, alginate hydrogel dressing-treated groups, alginate hydrogel/*Satureja khuzistanica* dressing-treated wounds, and control group. Exact studies of wound sites and the comparison of wounds showed that the healing process in alginate hydrogel/*Satureja khuzistanica* dressing-treated wounds was better than other groups. Also, the healing of alginate hydrogel/*Satureja khuzistanica* dressing-treated wounds was better than that of the *Satureja khuzistanica* and alginate hydrogel dressing-treated groups because of the keratinization of the epidermis. More progression in blood vessels formation was seen in experimental groups in comparison with the control group. The process of progression in granulation was observed in alginate hydrogel/*Satureja khuzistanica* dressing-treated wounds more than *Satureja khuzistanica*-treated wounds and alginate hydrogel dressing-treated groups. Our results demonstrated that there is no significant difference between the treated groups, but a significant difference was observed between the alginate hydrogel/*Satureja khuzistanica* dressing-treated wounds and control group.

### Light microscopic findings on the fourteenth day

All of the treated and control groups showed that wound healing and epidermis formation were on progress. Exact evaluations approved that the healing process of the samples of treated groups was better than the control group. Other observations displayed that the process of blood vessels formation continues especially in the treated groups. Our results approved that there is no significant difference between the treated groups, but a significant difference was observed between the treated groups and the control group.

### Light microscopic findings on the twenty-second day

Wound healing and epidermis formation and especially the thickness of the epidermis in treated wounds were more than that in the control group. The wound site was not clear and was improved, and growth of hair was completely visible in treated groups on the 22nd day of treatment. The formation of blood vessels decreased in the treated groups compared to that in the control group. The formation of granulation decreased in the treated groups compared to that in the control group.

### Histometric results

The obtained results of evaluating of wound healing percentage in skin tissue showed a significant increase in amelioration of skin wounds in the treated groups in comparison with the control group due to regenerative properties of *Satureja khuzistanica* extract and alginate hydrogel. The complete closure of the wound in the 22nd day and complete improvement of the wound on the 14th day were observed in the treated group. But, in the control group, complete improvement of the wound was seen after the 22 days. The obtained results of the assessment of the epidermis, blood vessels, and granulation amounts among the groups during the 3rd, 7th, 14th, and 22nd days have been presented as follows.

#### Comparison of wound epidermis scores during the 3rd, 7th, 14th, and 22nd days

**Fig. 1:**
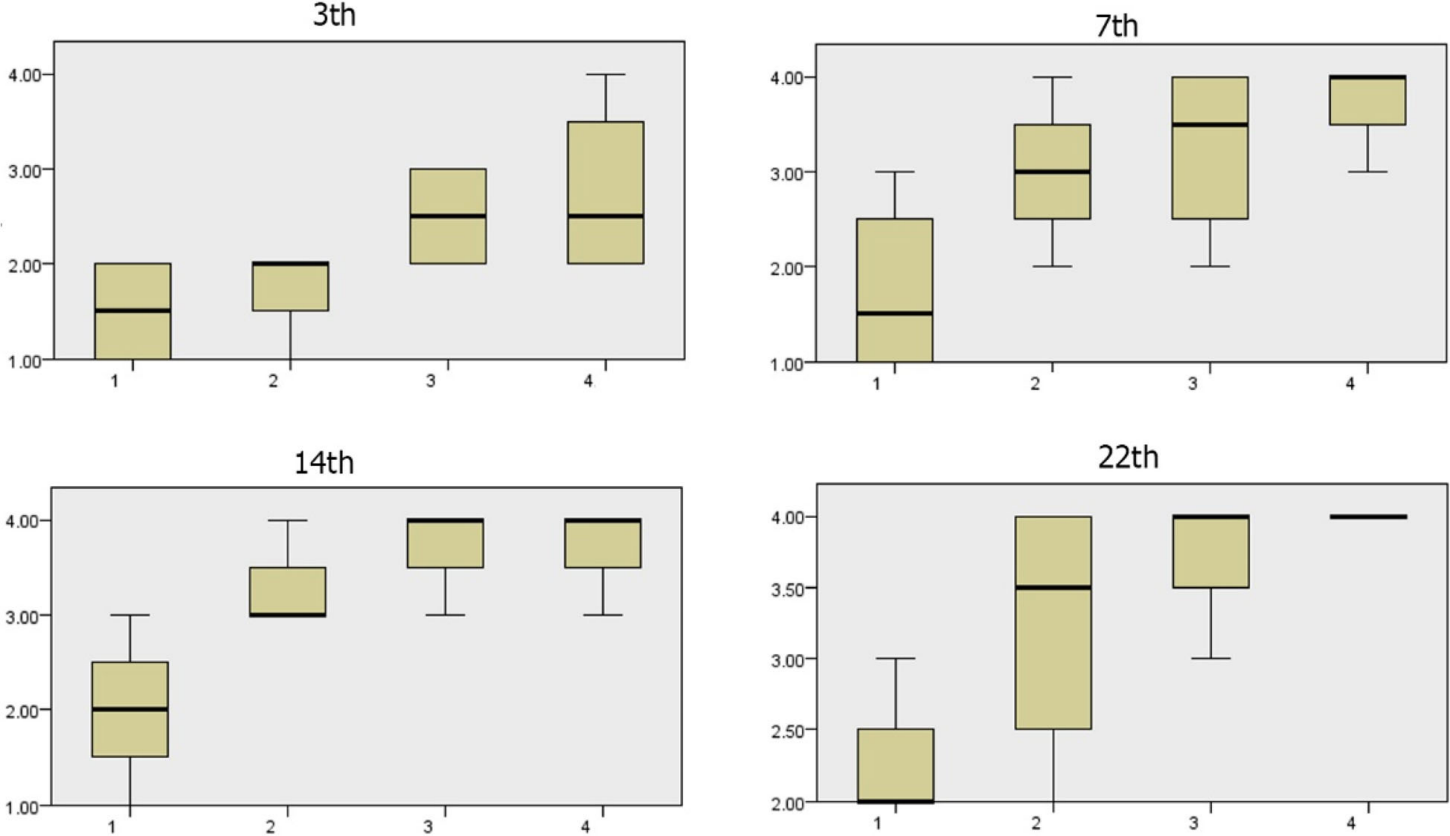
Wound epidermis scores during the 3rd, 7th, 14th, and 22nd days. There is a significant difference in the amount of formation of wound epidermis during the 3rd, 7th, 14th, and 22nd days in groups, and more epidermis formation causes more improvement. The amount of wound epidermis formation in the treated groups was more than the control group. (1) Control group, (2) *Satureja khuzistanica*-treated group, (3) alginate hydrogel dressing-treated group, and (4) alginate hydrogel/*Satureja khuzistanica* dressing-treated group

According to Fig. 1, the amount of wound epidermis scores has increased in treated groups on the 3rd, 7th, 14th, and 22nd days compared to that in the control group. There is a significant difference in wound epidermis scores during the 3rd, 7th, 14th, and 22nd days in each of the four groups (*p* < 0.05). Wound epidermis scores in the alginate hydrogel/*Satureja khuzistanica* group were more than that in the other treated groups and control group on the 7th day. In this comparison, more epidermis formation causes more improvement. The amount of formation of wound epidermis in the alginate hydrogel/*Satureja khuzistanica* and alginate hydrogel groups were more than that in *Satureja khuzistanica* group and control group on the 14th day. In this comparison, more epidermis formation causes more improvement. The amount of wound epidermis formation in the treated groups was more than that in the control group.

#### Comparison of wound blood vessels scores during 3rd, 7th, 14th, and 22nd days

**Fig. 2:**
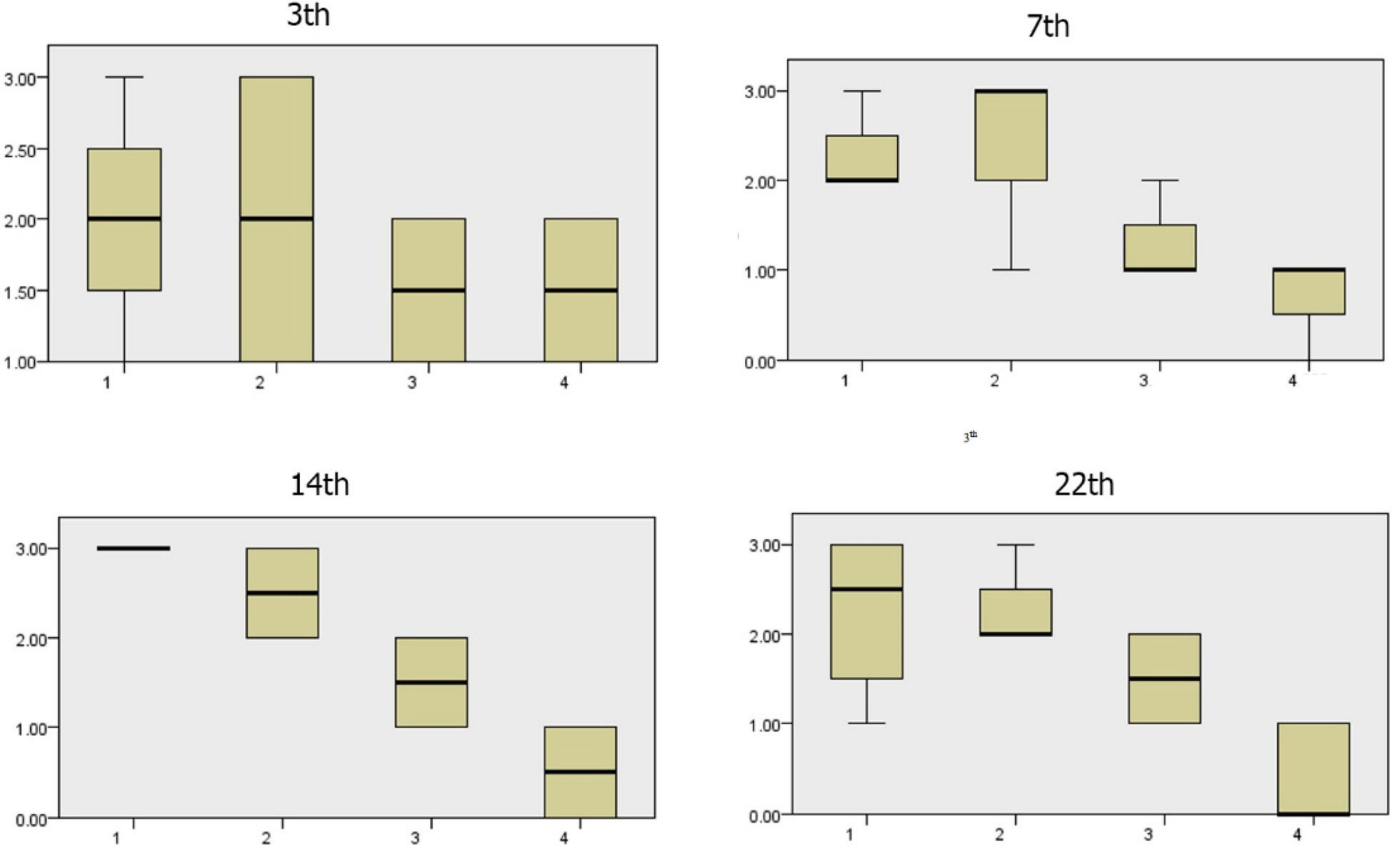
Wound blood vessels scores during the 3rd, 7th, 14th, and 22nd days. There is a significant difference in the amount of wound blood vessel formation. In the treated groups, the amount of wound blood vessels formation was less than the control group, and less blood vessel formation causes more improvement to lead to effects in the healing process more than the control group. (1) Control group, (2) *Satureja khuzistanica*-treated group, (3) alginate hydrogel dressing-treated group, and (4) alginate hydrogel/*Satureja khuzistanica* dressing-treated group

According to Fig. 2, the amount of wound blood vessels during the 3rd and 22nd days were more than that during the 7th and 14th days in the treated groups. Also, the amount of wound blood vessels in the control group was more than that in the treated groups on the 22nd day. This decreased amount of wound blood vessels in the treated group on the 22nd day shows positive involving of treatment in the healing process in comparison with the control group. There is a significant difference in wound blood vessels scores during the 3rd and 22nd days (*p* < 0.05). The amount of wound blood vessels formation in the treated groups was more than that in the control group on the 3rd day. It seems that the alginate hydrogel-treated group’s effect in the healing process was more than those of the other treated groups and control group. The amount of wound blood vessel formation in the treated groups was less than that in the control group on the 22nd day. In this comparison, less blood vessel formation causes more improvement. It seems that all of the treated groups’ effects in the healing process were more than that of the control group on the 22nd day.

#### Comparison of granulation scores during 3rd, 7th, 14th, and 22nd days

**Fig. 3.**
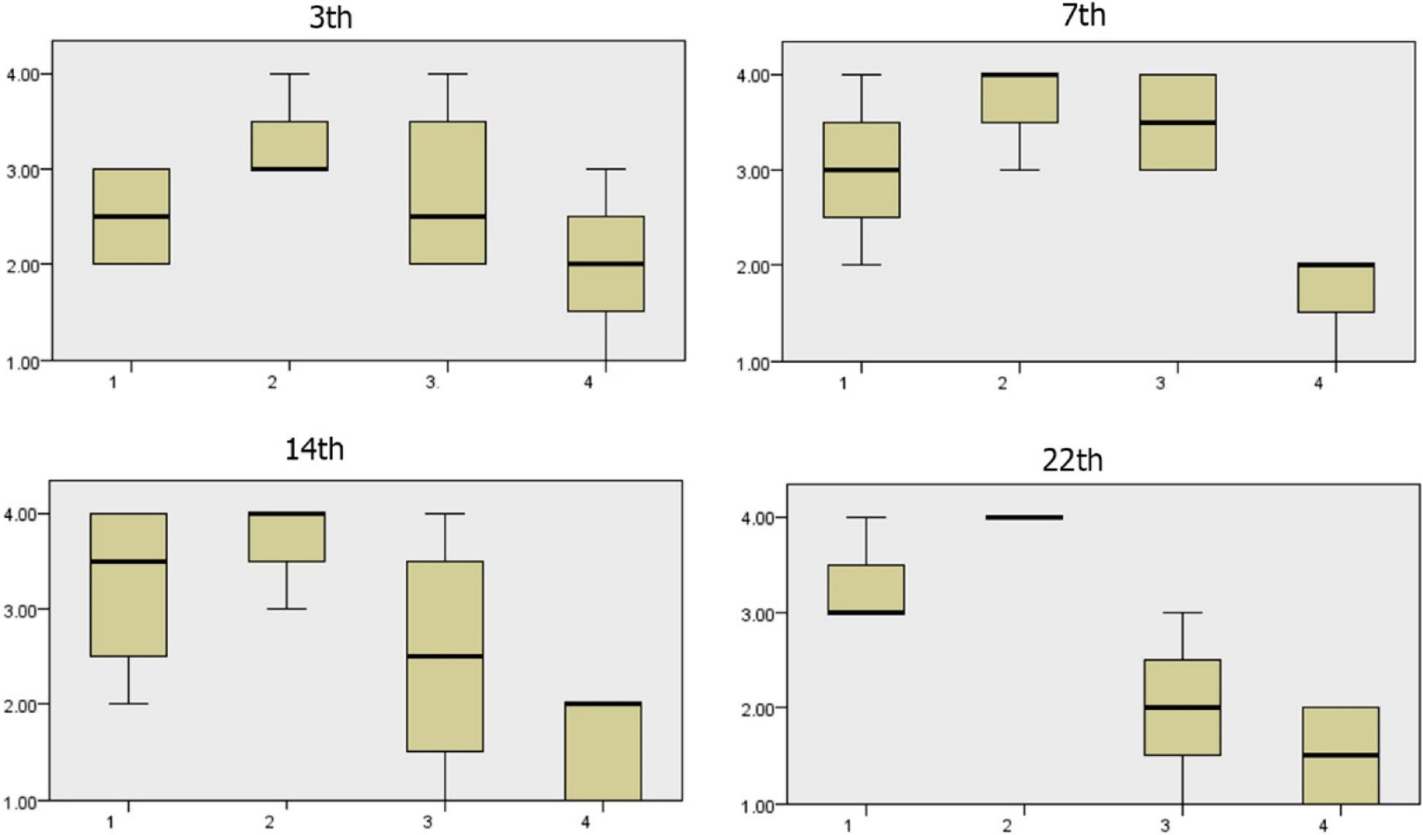
Granulation scores during the 3rd, 7th, 14th, and 22nd days. There is a significant difference in the amount of wound granulation during the 7th and 14th days between the control and treatment groups. The amount of wound granulation formation in the *Satureja khuzistanica*-treated group was more than that in the other treated groups and control group on the 14th day. (1) Control group, (2) *Satureja khuzistanica*-treated group, (3) alginate hydrogel dressing-treated group, and (4) alginate hydrogel/*Satureja khuzistanica* dressing-treated group

**Fig. 4:**
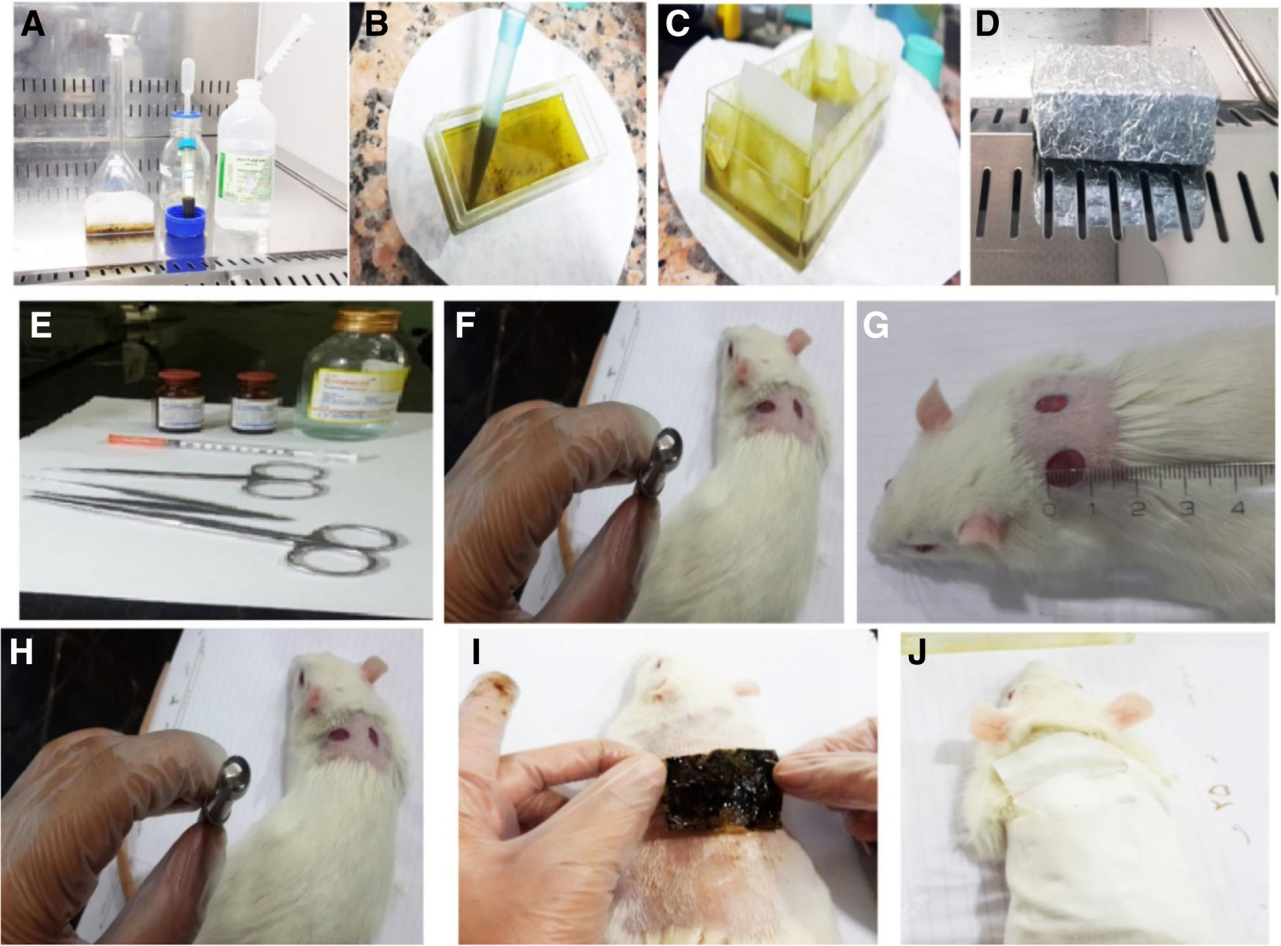
**a**–**d** Preparation of alginate hydrogel and *Satureja khuzistanica*. **e**–**h** The rats were prepared for surgery and the wound was cut in the form of 1 cm circular diameter by using biopsy punch in three layers of the skin (dermis, epidermis, and hypodermis). **i**, **j** Wound along with alginate hydrogel dressing and ethanolic extract of *Satureja khuzistanica* treatment

**Fig. 5:**
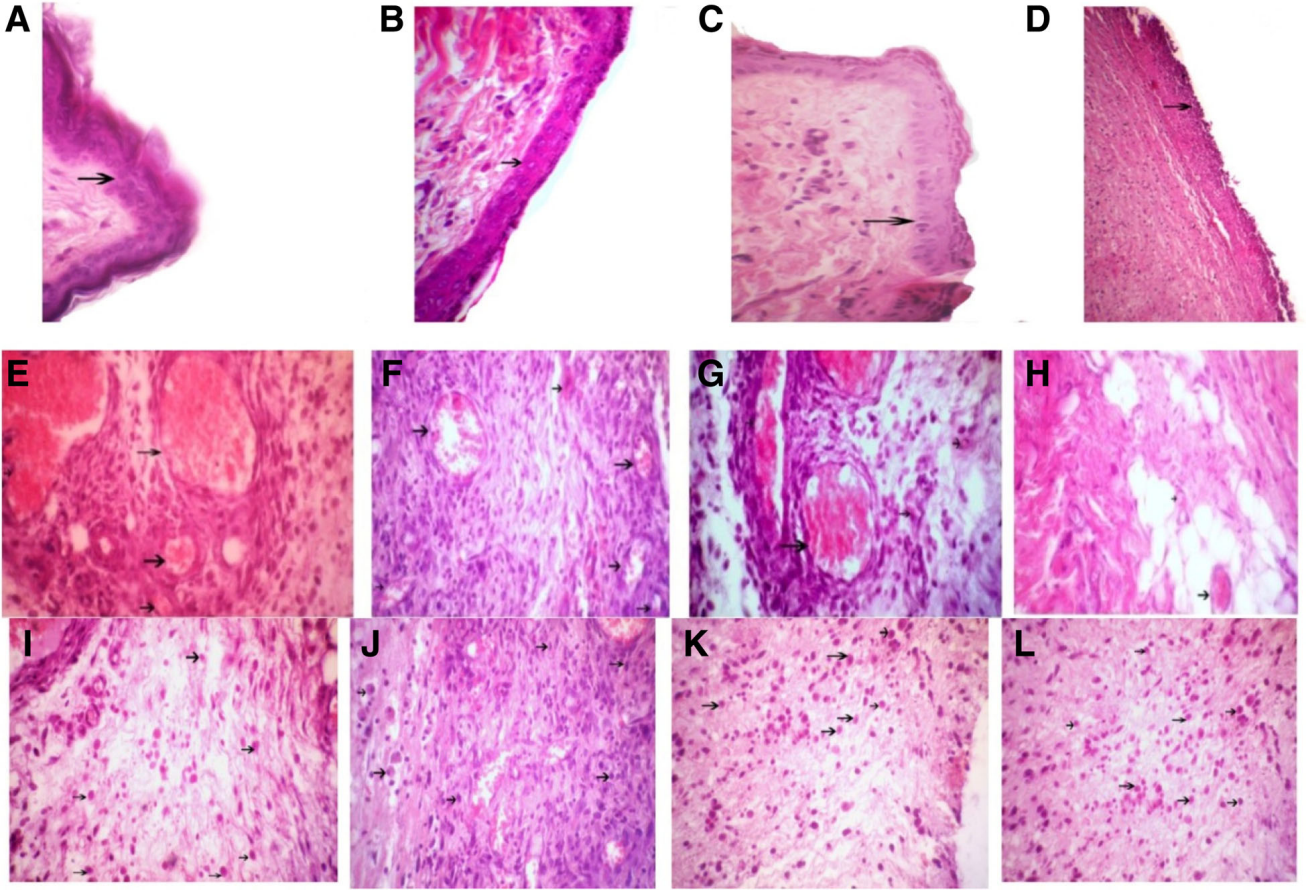
Arrows show the wound epidermis regeneration (**a–d**), vascular formation (**e**–**h**), and granulation (**i–l**) on the third day. **a**, **e, i** *Satureja khuzistanica*-treated group. **b**, **f**, **j** Hydrogel alginate-treated group. **c**, **g**, **k** *Satureja khuzistanica* encapsulated in hydrogel alginate-treated group. **d**, **h**, **l** Control group. Hematoxylin and eosin stain (× 400). Arrows show the re-epithelialization with mild hyperplasia of the epidermis and moderate hyperkeratosis (**a**–**d**) and immature granulation tissue (**i**–**l**) with emerging blood vessels (angiogenesis) shown in black arrows and fibroblasts and numerous macrophages

**Fig. 6:**
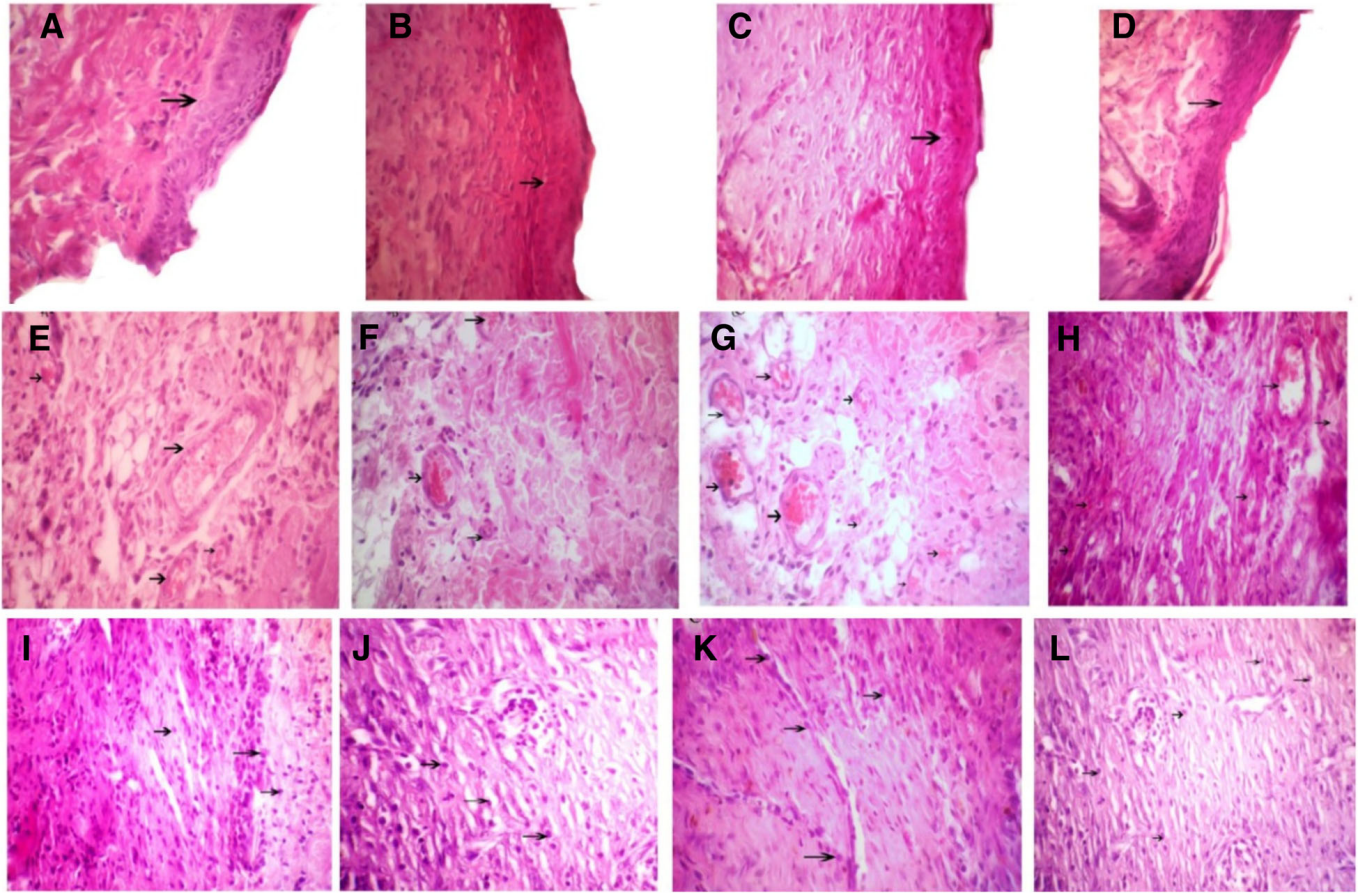
Arrows show the wound epidermis regeneration (**a–d**), vascular formation (**e–h**), and granulation (**i–l**) on the seventh day. **a**, **e**, **i** *Satureja khuzistanica*-treated group. **b**, **f**, **j** Hydrogel alginate-treated group. **c**, **g**, **k** *Satureja khuzistanica* encapsulated in hydrogel alginate-treated group. **d**, **h**, **l** Control group. Arrows show the re-epithelialization with mild hyperplasia of the epidermis and moderate hyperkeratosis (**a**–**d**) and immature granulation tissue (**i**–**l**) with emerging blood vessels (angiogenesis) shown in black arrows and fibroblasts and numerous macrophages. Hematoxylin and eosin stain (× 400)

**Fig. 7:**
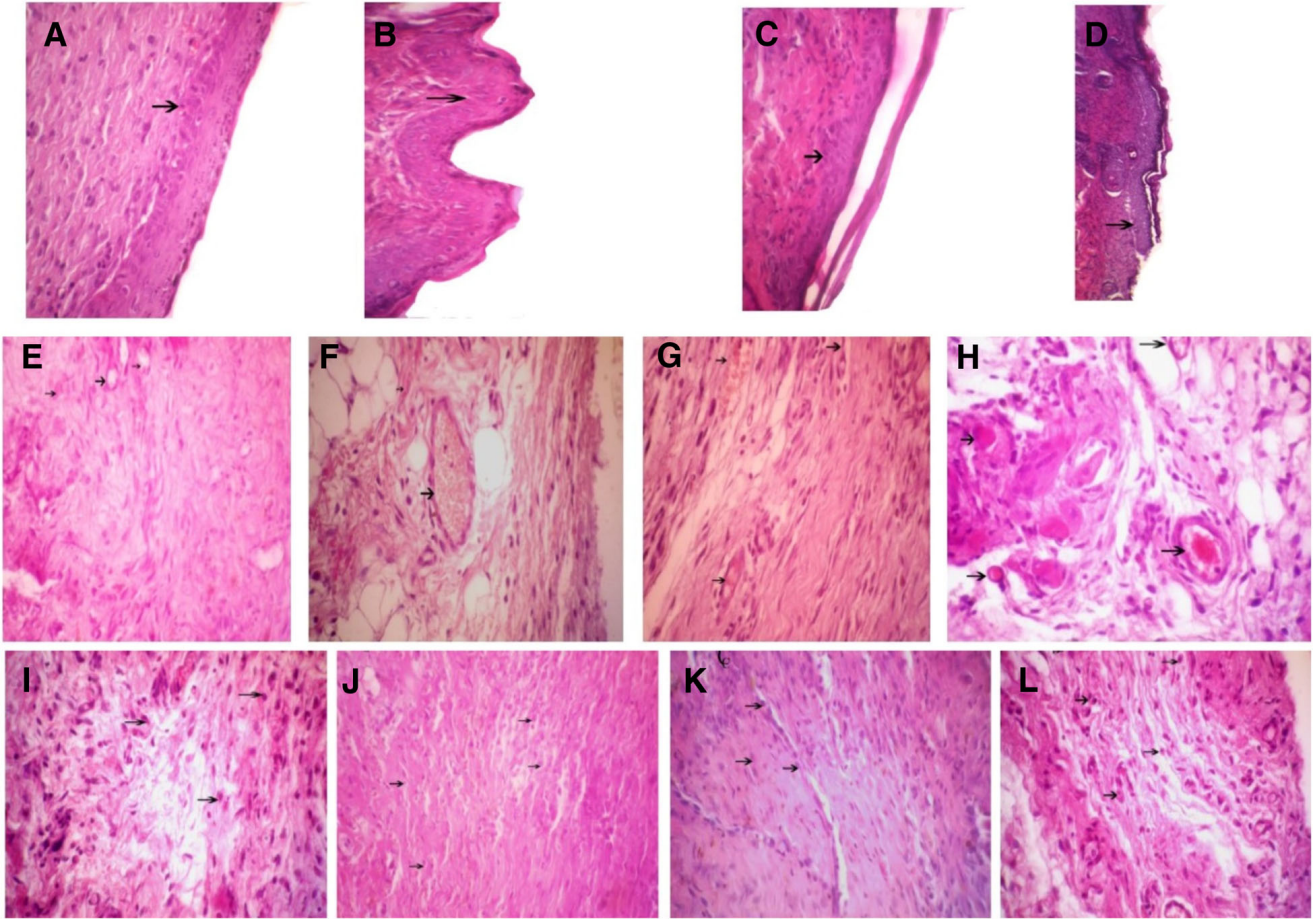
Arrows show the wound epidermis regeneration (**a–d**), vascular formation (**e–h**), and granulation (**i–l**) on the 14th day. **a**, **e**, **i** *Satureja khuzistanica*-treated group. **b**, **f**, **j** Hydrogel alginate-treated group. **c**, **g**, **k** *Satureja khuzistanica* encapsulated in hydrogel alginate-treated group. **d**, **h**, **l** Control group. Hematoxylin and eosin stain (× 400). Arrows show the re-epithelialization with mild hyperplasia of epidermis and moderate hyperkeratosis (**a**–**d**) and immature granulation tissue (**i**–**l**) with emerging blood vessels (angiogenesis) shown in black arrows and fibroblasts and numerous macrophages

**Fig. 8:**
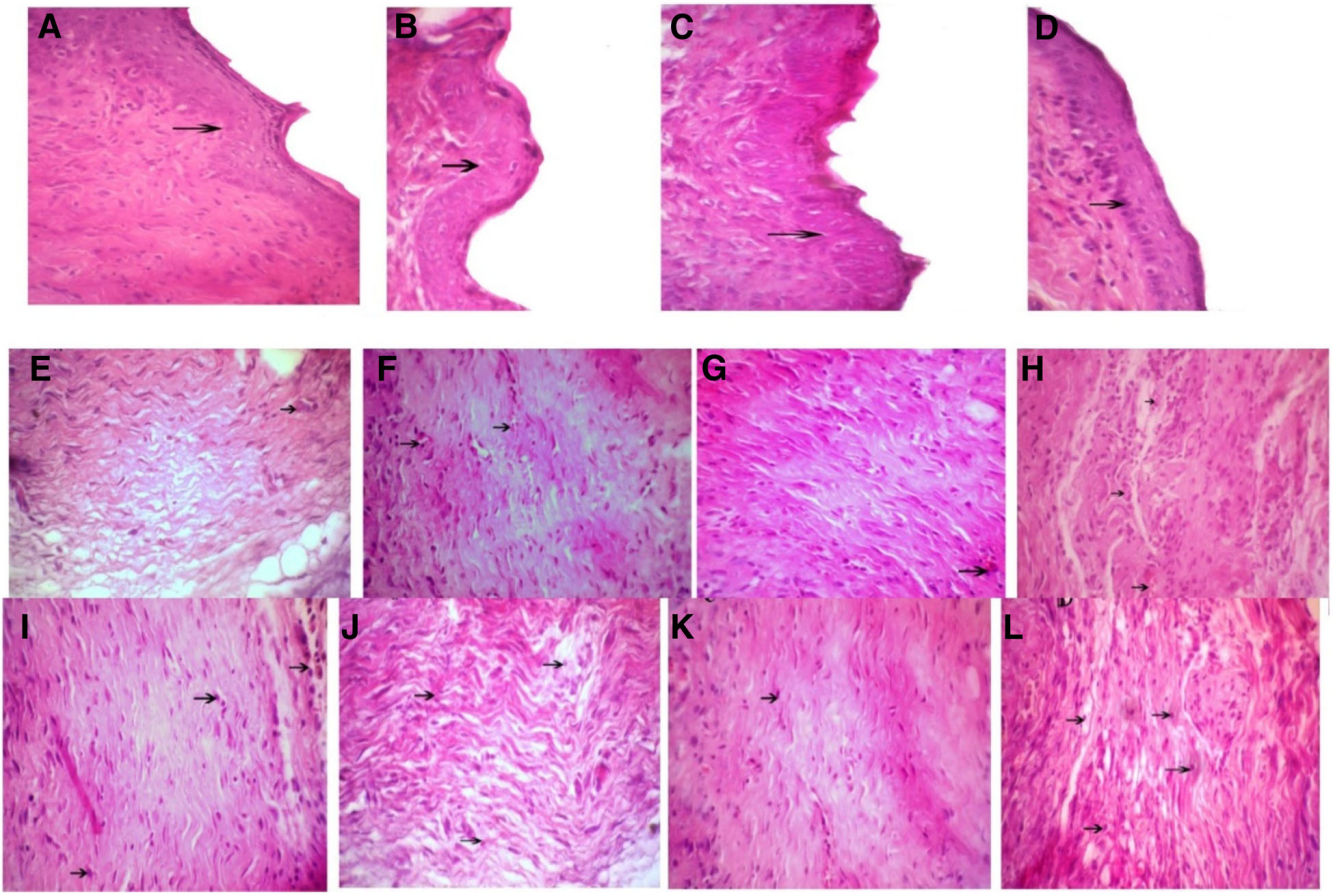
Arrows shows wound epidermis regeneration (**a–d**), vascular formation (**e–h**), and granulation (**i–l**) on the 22nd day. **a**, **e**, **i** *Satureja khuzistanica*-treated group. **b**, **f**, **j** Hydrogel alginate-treated group. **c**, **g**, **k** *Satureja khuzistanica* encapsulated in hydrogel alginate-treated group. **d**, **h**, **l** Control group. Hematoxylin and eosin stain (× 400)

**Fig. 9:**
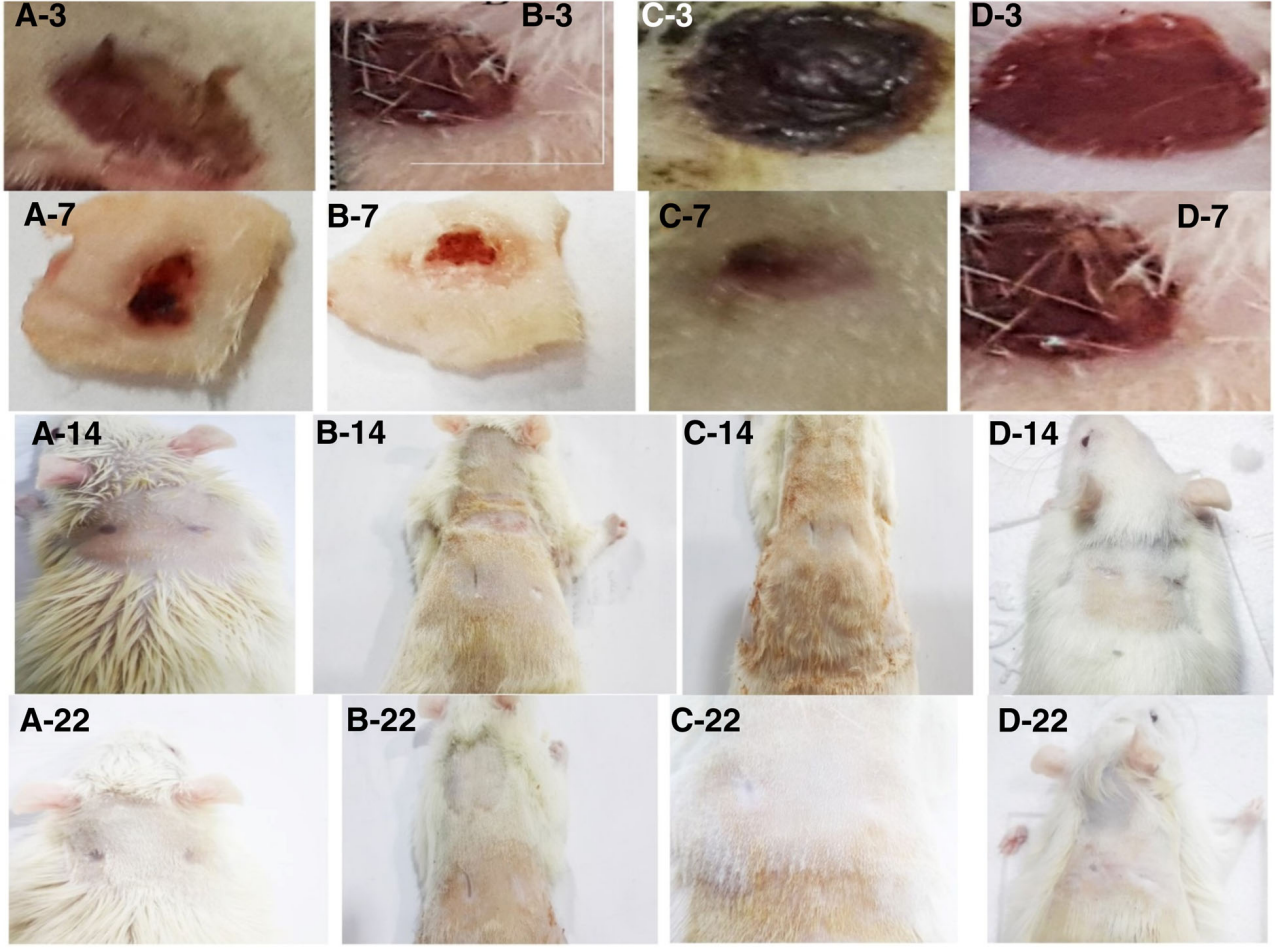
The appearance of wound healing in different treated groups. **a–d** show the *Satureja khuzistanica*-treated, alginate hydrogel dressing-treated, alginate hydrogel/*Satureja khuzistanica* dressing-treated, and control groups, respectively. The numbers indicate the number of days of treatment

The amount of wound granulation during the 7th and 14th days was more in the control group (Figs 3, 4, 5, 6, 7, 8, and 9). The amount of wound granulation formation in the alginate hydrogel/*Satureja khuzistanica*-treated group was more than that in the other treated groups and control group on the 7th day. The amount of wound granulation formation in the *Satureja khuzistanica*-treated group was more than that in the other treated groups and control group on the 14th day.

## Discussion

Wound healing is recognized as a complicated process, as mentioned earlier. It is consisting of many circumstances that involve cellular and biochemical events. The series of events involved in the wound healing are as follows: inflammation, migration of the cells, proliferation, and remodeling. Briefly, vasoconstriction, activation of coagulation process, infiltration of immune cells, and secretion of cytokines are the common events that occur during inflammatory phase. In the migration phase, inflammatory cells especially neutrophils and macrophages migrate to the wound site. Angiogenesis and proliferation of fibroblasts and epithelial cells are the main processes that happen in the proliferation phase. Finally, fibroblasts secrete collagen in the wound site and a new tissue was generated [3, 24]. Selection of a suitable dressing is a good choice for the treatment of wounds [25]. An appropriate dressing should provide moisture for the development of better wound healing [26]. Hydrogels as a biomaterial with a network structure can help supply wound moisture through water retention. Hence, it can play an important role in wound healing [27]. Alginate is another biomaterial that improves wound healing via moisture supply and absorption of wound exudate [28]. Thus, according to the properties of these biomaterials, the combination of them together with medicinal plants is a suitable solution for wound healing. *Satureja khuzistanica* is one of these herbs that can be useful because its antioxidant, anti-inflammatory, and antimicrobial properties can be useful in the wound healing process [29].

So far, many studies were conducted about the antioxidant properties and therapeutic effects of *Satureja khuzistanica*. But the therapeutic effect of *Satureja khuzistanica* extract encapsulated in alginate hydrogels on cutaneous wound healing in rats was not studied. So, we decided to evaluate the restorative effect of *Satureja khuzistanica* extract encapsulated in alginate hydrogels on cutaneous wound healing in rats. The histological results of the present study showed that topical administration of ethanolic extract of *Satureja khuzistanica* encapsulated in alginate hydrogels has a beneficial effect on wound healing rate via improvement in some parameters including increased epidermis formation and reduced granulation and angiogenesis on the 3rd, 7th, 14th, and 22nd days compared to the control group. So, the amount of epidermis formation was more increased in the treated groups compared to that in the control group on the 7th, 14th, and 22nd days. There was a significant difference between the treated groups and the control group. But, this difference was not significant on the 3rd day. In addition, complete hair growth was observed on day 22. Epithelialization is one of the most important stages in wound healing process. It starts with the migration of healthy epithelial cells from the edges of the wound and finally covers all of the wound [30]. A related study demonstrated that the ethanolic extract of *Satureja rechingeri* has a positive role in wound healing via increased epithelial regeneration on the 7th and 14th days [5]. Moreover, Murakami et al. displayed that the hydrogel blends of chitin/chitosan, fucoidan, and alginate significantly increased re-epithelialization on the 7th day [31]. Another study showed that wound healing can be increased by using silk fibroin/alginate-blended sponge through increased size of re-epithelialization and epithelial cells [32]. Balakrishnan et al. supported the positive role of alginate dialdehyde cross-linked gelatin hydrogels in enhanced epithelial cell migration during the healing process [33]. More studies including the study of Lee et al. demonstrated that layered hydrogel composing of alginate (AL), chitosan (CS), and poly(gamma-glutamic acid) (PGA) can enhance epithelialization in the wound site [34]. The results of the above studies are consistent with the results of our study.

The number of blood vessels has a significant difference in the treated groups in comparison with the control group on the 3rd and 22nd days. At the beginning of the healing phase, the number of blood vessels was increased, but at the end of this period, it decreased, which is consistent with this phase. The phenomenon of angiogenesis plays a vital role in wound healing. It is demonstrated that wound tissue nutrition and protection of the cells involved in wound healing such as macrophages and fibroblasts through oxygen delivery are the most important tasks of angiogenesis in the wound healing process [35]. Gholami et al. showed that ethanolic extract of *Satureja rechingeri* can participate in the wound healing process through decreased angiogenesis on the 7th and 14th days [5]. A similar research confirmed that hydrogel blends of chitin/chitosan, fucoidan, and alginate can stimulate angiogenesis on the 7th day and subsequently wound healing [31]. The results of the above studies are consistent with the results of our study.

The results showed that granulation of tissue at days 7 and 14 is a significant difference compared to that of the control group. Moreover, the results showed that the granulation of tissue has a significant difference on 7th and 14th days compared to the control group. Tissue granulation was decreased at the end of the remodeling process. Granulation is another factor that affects the healing process. Combination of blood vessels with migratory cells in the wound site create granulation tissue that during the first week of scar formation reaches its maximum [36]. Gholami et al. showed that ethanolic extract of *Satureja rechingeri* can play a beneficial role in the wound healing process through decreased tissue granulation on the 7th and 14th days [5]. Another research confirmed that hydrogel blends of chitin/chitosan, fucoidan, and alginate involve in the wound healing by advanced tissue granulation on the 7th day [31]. A related study demonstrated that alginate dialdehyde cross-linked gelatin hydrogels can develop an appropriate environment for tissue granulation [33]. The results of the above studies are consistent with the results of our study.

Other studies suggested that some biomaterials including hydrogel films composed of alginate and *Aloe vera* gel and alginate–hyaluronan composite hydrogels can cause acceleration of wound healing process [14, 37].

## Conclusion

According to the results, the effectiveness of the encapsulated extract of *Satureja khuzistanica* on wound healing in hydrogel alginate was demonstrated. It is possible that this property returns the presence of bioactive compounds and antioxidants in *Satureja khuzistanica* and also the unique properties of hydrogel alginate dressing including biocompatibility and hydrophilic, preparing a moist environment. In addition, *Satureja khuzistanica*/hydrogel alginate dressing can be easily separated without pain.
